# Genetic relatedness among isolates of *Shigella sonnei *carrying class 2 integrons in Tehran, Iran, 2002–2003

**DOI:** 10.1186/1471-2334-7-62

**Published:** 2007-06-22

**Authors:** Reza Ranjbar, Aurora Aleo, Giovanni M Giammanco, Anna Maria Dionisi, Nourkhoda Sadeghifard, Caterina Mammina

**Affiliations:** 1 Research Center of Molecular Biology, Baqiyatallah University of Medical Sciences, Tehran, Iran; 2 Dipartimento di Igiene e Microbiologia "G. D'Alessandro", Università di Palermo, Via del Vespro 133, I-90127 Palermo, Italy; 3 Dipartimento di Malattie Infettive, Parassitarie e Immunomediate, Istituto Superiore di Sanità, Viale Regina Elena 299, I-00161Rome, Italy; 4 Department of Microbiology, Faculty of Medicine, Ilam University of Medical Sciences, Ilam, Iran

## Abstract

**Background:**

*Shigella *spp. are major cause of diarrhoeal disease in both developing and developed countries. *Shigella sonnei *is the serogroup of *Shigella *most frequently responsible for sporadic and epidemic enteritis in developed countries. In recent years the emergence and spread of *S. sonnei *biotype g carrying class 2 integron have been frequently reported in many countries. Recently, *S. sonnei *has been reported as the prevalent serogroup of *Shigella *in Iran.

The present study was carried out to investigate phenotypic and genetic characteristics of *Shigella sonnei *isolates identified in the years 2002 and 2003 in Tehran, Iran.

**Methods:**

Biotyping, drug susceptibility testing, pulsed field gel electrophoresis (PFGE) and analysis of class 2 integrons have been carried out on 60 *S. sonnei *isolates, including 57 sporadic isolates from paediatric cases of shigellosis occurring in 2002 and 2003, two sporadic isolates recovered in 1984 and the ATCC 9290 strain.

**Results:**

Biotype g and resistance to streptomycin, sulfamethoxazole-trimethoprim and tetracycline were exhibited by 54 of the 57 recent isolates. Of the 54 biotype g isolates, 28 exhibited a class 2 integron of 2161 bp, and 24 a class 2 integron of 1371 bp, respectively. Class 2 integrons were not detected in four isolates only, including the two endemic isolates recovered in 1984 and two strains from recent sporadic cases. PFGE divided the strains into eight pulsotypes labeled A to H, three major pulsotypes – A to C – including the large majority of the recent sporadic *S. sonnei *isolates. Pulsotypes A and C were the most prevalent groups, accounting for 41.6% and 35.0%, respectively, of the isolates under study.

**Conclusion:**

The results suggest that biotype g, class 2 integron carrying *S. sonnei *are prevalent in our geographic area. *S. sonnei *isolated in the years 2002 and 2003 could be attributed to a few predominant clusters including, respectively, strains with pulsotypes B and C carrying a 2161 bp class 2 integron, and those having pulsotype A and a 1371 bp class 2 integron. A few epidemic clones are responsible for the apparently endemic occurrence of shigellosis in Tehran, Iran.

## Background

Infections caused by *Shigella *species are a major cause of diarrhoeal disease in both developing and developed countries. Globally, it is estimated that shigellosis causes about 1,100,000 deaths per year, two-thirds of the patients being children under 5 years of age [1,2].

Development and spread of multidrug resistance is becoming a serious threat to Public Health worldwide since the early 1960s [3]. Mobile genetic elements, such as plasmids, transposons, genomic islands and integrons, disseminating via horizontal transfer are exponentially amplifying the problem [4]. In particular, the role of integrons in the dissemination of multidrug resistance in Gram-negative bacteria is well-established [5], and their carriage by several members of the family *Enterobacteriaceae *has been reported [6].

Recently, several reports have shown the successful emergence and spread in different countries of *S. sonnei *biotype g carrying a class 2 integron [4,7-12]. Pulsed Field Gel Electrophoresis (PFGE) analysis has proved that related groups of isolates may have circulating in recent years in different European countries [8]. Emergence and selective pressure by use of some antimicrobial agents or, alternatively, international trade are under debate as possible epidemiological mechanisms of the successful dissemination of this organism [13].

Shigellosis is one of the major causes of morbidity in children with diarrhea in Iran, but reports about prevalence of serogroups and molecular epidemiological features are limited. In two previous studies, *S. flexneri *has been reported as the most prevalent species in this country in the years 1984–'85 and 2001–'02 [14,15]. Similar patterns of serogroup prevalence have been detected in some neighboring countries. Indeed, *S. flexneri*, as the most frequent serogroup, accounted for 44.0%, 58.0%, and 65.0%, respectively, of shigellosis cases in Saudi Arabia [16], Pakistan [17] and Jordan [18]. In a subsequent investigation conducted in 2003, however, shifting of species distribution of *Shigella *appeared to occur in Tehran, Iran, where the proportion of *S. sonnei *isolates was 58.9 %, while that of *S. flexneri *36.4% [19]. More recently, Farshad et al. (2006) has identified *S. sonnei *as the most prevalent *Shigella *species in Shiraz, Iran [20].

The present study was therefore undertaken to describe phenotypic and genetic characteristics of *S. sonnei *isolates identified in the years 2002–2003 in Tehran, Iran. Prevalence of biotypes, drug susceptibility patterns, pulsotypes and carriage of class 2 integrons was investigated with special attention to obtain an interpretative key of the apparently endemic occurrence of disease in this geographic area.

## Methods

### Bacterial strains

In the years 2002 and 2003, a total of 178 isolates of *S. sonnei *have been isolated from enteritis cases in children at five hospitals in Tehran, Iran. A random sample of 57 isolates was selected for the investigation. Three further strains were added: the *S. sonnei *strain ATCC 9290 and two apparently sporadic strains isolated in Tehran, Iran, in 1984.

All strains had been identified at a genus level by conventional methods by previously described procedures [21] while agglutination with specific antiserum from MAST Group LTD (Mast House, Derby Road, Bootle, Merseyside, L20 1EA, UK) was used to identify the species.

Biotyping was performed by the method of Nastasi et al. [21]. Antimicrobial susceptibility test was performed by the disk diffusion method according to the guidelines of the National Committee for Clinical Laboratory Standards [22]. The following antimicrobial agents were tested: ampicillin, AMP (10 μg); ceftriaxone, CRO (30 μg); kanamycin, K (30 μg); streptomycin, STR (10 μg); tetracycline, TET (30 μg); ciprofloxacin, CP (5 μg); nalidixic acid, NA (30 μg); chloramphenicol, C (30 μg); and sulfamethoxazole-trimethoprim, SXT (1.25/23.75 μg). *Escherichia coli *ATCC 25922 was used as a quality control strain.

### Pulsed Field Gel Electrophoresis (PFGE) analysis

All *S. sonnei *isolates were analyzed by PFGE with the restriction enzyme *Xba*I (Promega, Madison, WI, USA) under standardized conditions [23]. The *Salmonella *serotype Braenderup strain H9812 restricted with *Xba*I was used as molecular weight standard. Strain H9812 was kindly provided by the National Reference Centre for Enteric Pathogens at Istituto Superiore di Sanità, Rome, Italy.

PFGE patterns were initially visually assessed and interpreted by using the criteria established by Tenover et al. [24]. Computer-assisted analysis of the PFGE banding patterns was then performed with the Taxotron software (Taxolab, Institut Pasteur, Paris, France). Genetic distances were calculated using the Unweighted Pair Group Method using arithmetic Averages (UPGMA). Only bands whose molecular weight exceeded 90 Kbp were considered and the calculated error was set at 10% for low molecular weight and progressively decreased to 5% for the highest weights (1,000 Kbp).

### Class 2 integron analysis

To detect the class 2 integrons, primer pair hep74 (5'-CGGGATCCCGGACGGCATGCAC GATTTGTA-3') and hep51 (5'-GATGCCATCGCAAGTACGAG-3') was used under conditions described previously [8]. Amplified DNA products of interest were cloned by use of the TA cloning Kit (Invitrogen Life Technology, Italy) in invα F' cells. The cloned products were sequenced by fluorescent dye-labeled dideoxynucleotides and a 373 automatic DNA sequencer (Perkin-Elmer, Foster City, CA), using the universal primers M13fw and M13rev for the first 1400 bp and the primer ss1, 5'-AAGTGGCAGCAACGGATTCG-3', to complete the sequence. The resulting DNA sequence was analyzed by use of the BLAST search program 2.0 within the QBLAST system at the National Center for Biotechnology Information site.

### PFGE-CeuI and hybridization

To establish if the class 2 integrons were located on the chromosome, the restriction enzyme *Ceu*I was used to generate a pulsed-field gel electrophoresis (PFGE) profile to be hybridized with the *int*2 sequence. *Ceu*I is a rare-cutting enzyme, which produces very large restriction fragments. The preparation of genomic DNA of *S. sonnei *strains was performed according to the above protocol. Then, DNA restriction was done with 10U *Ceu*I enzyme (New England Biolabs, Beverly, MA, US) at 37°C for 3 h and DNA macrorestriction fragments were resolved on 0.7% agarose gel (Pulsed Field Certified, Bio-Rad, Hercules, CA, US) prepared in 0.5X TBE buffer (50 mM Tris, 50 mM boric acid, 0.5 mM EDTA). Lambda ladder concatamers (New England Biolabs) were used as molecular marker. The gel was run on a CHEF-DRII system (Bio-Rad Laboratories) under the conditions described by Liu *et al*. [25]. The gel, after staining for 10 min in ethidium bromide solution (0.4 μg/ml) and destaining in distilled water for 20 min, was visualised under UV light and photographed.

Restricted fragments were transferred onto positively charged nylon membranes (Roche Diagnostics, Monza, Italy) by standard methods [25]. Southern blot hybridization was carried out under high-stringency conditions [26] using specific *int*2 and 16sRNA probes, obtained amplifying the internal part of the respective genes with the following primers: *int2*, ssfw 5'-TTTCAGGTGGTGGGGAGATA-3' and ssrv 5'-TTGGTACAAAAGGCGTGACA-3'; 16sRNA, S16fw 5'-CAGCCACAC-TGGAACTGAGA-3' and S16rev 5'-GTTAGCCGGTG-CTTCTTCTG-3'. Probes were labelled by the "PCR DIG labelling" kit (Roche Diagnostics).

## Results

Fifty-four of the 57 isolates of *S. sonnei *identified in the period 2002–'03 and selected for the study were attributed to biotype g and three only to biotype a. Both strains isolated in 1984 were biotype g (Additional file 1).

Additional file 1 summarizes the antimicrobial resistance phenotypes of the isolates under study. Fifty-four *S. sonnei *isolates obtained during the years 2002 and 2003 were resistant to streptomycin, sulfamethoxazole-trimethoprim and tetracycline. Additional resistances to nalidixic acid, ampicillin or kanamycin were carried by four, five and two isolates, respectively. Two further isolates showed ampicillin resistance associated to nalidixic acid or kanamycin, respectively. No isolates were resistant to ceftriaxone, ciprofloxacin, or chloramphenicol.

**Figure 1:**
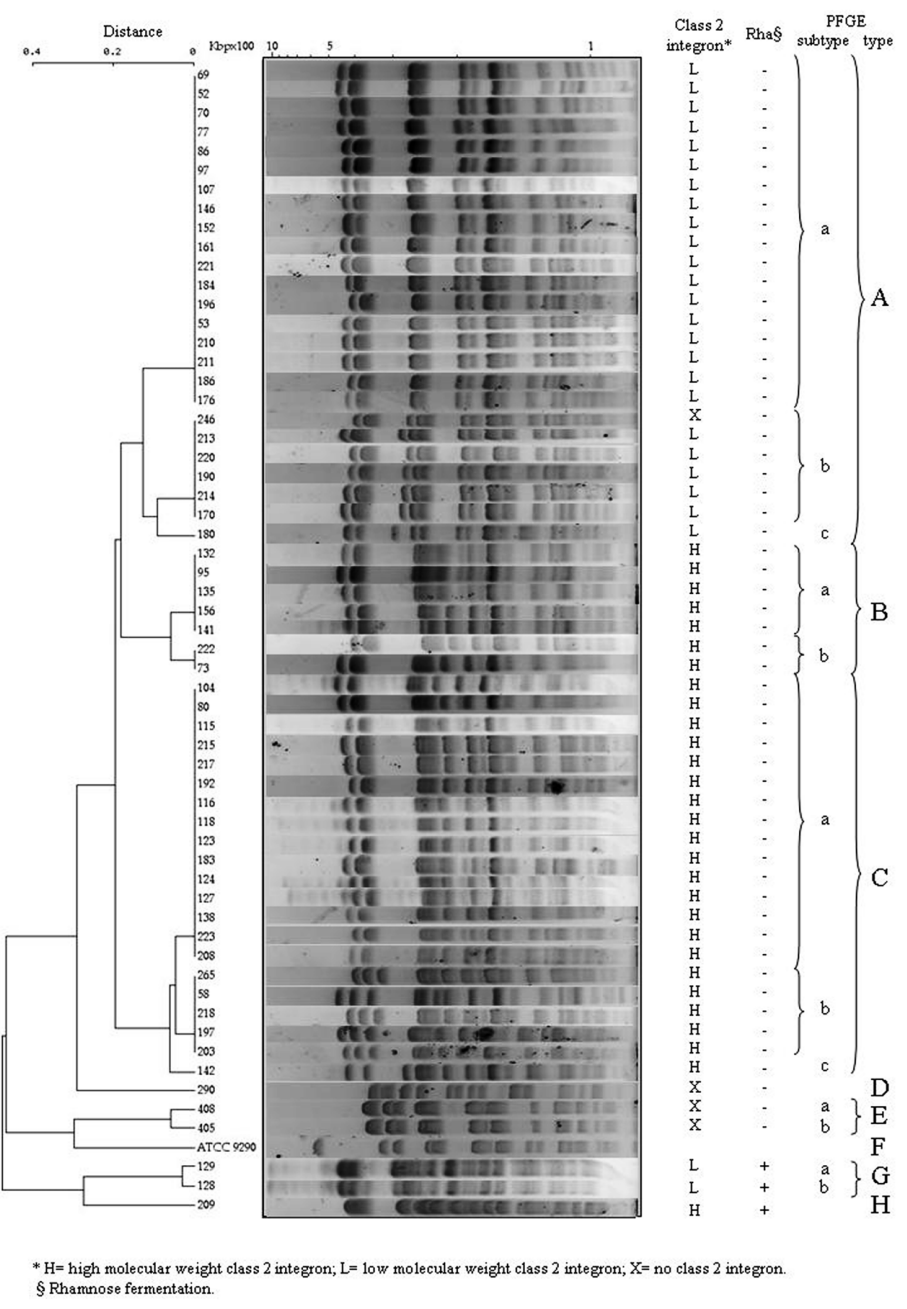
Phylogenetic dendrogram showing the genetic distances obtained from th comparison of the PFGE patterns produced using *Xba*I restriction endonuclease from 59 *Shigella sonnei *isolates and from the type strain of the serogroup.

Three major clusters of *S. sonnei *were identified by *Xba*I-PFGE among the sample of isolates recovered in Tehran, Iran, in 2002 and 2003 (Figure 1): PFGE type A (*n *= 25 isolates), PFGE type B (*n *= 7 isolates) and PFGE type C (*n *= 21 isolates). Three subtypes – a to c – were included into PFGE type A, subtype a including 18 of the 25 strains. Two subtypes, a and b, grouping five and two isolates, respectively, were observed among type B strains. Type C contained three subtypes, a to c, but subtype a included 15 of 21 isolates. The two isolates identified in 1984 were type E.

Two different class 2 integron structures were identified within the isolates under study. Twenty-eight (49.1%) of the 57 isolates shared a 2161 bp class 2 integron, identified by DNA sequencing as containing four open reading frames, namely *dhfrI*, *satI*, *aadA *and *orfX *(99% of homology with respect to the class 2 integron described by Pan JC *et al*, 2006). The second smaller integron of 1371 bp containing only two genes within the cassettes, *dhfrI *and *satI*, was detected in 24 strains (42.1%). Alignments of the deduced amino acid sequences revealed two sequence substitutions in both gene cassettes, *dhfrI *and *satI*. The sequence of the class 2 integron of 1371 bp has been submitted to GenBank (accession number AM745943).

Class 2 integrons were not detected in two strains from sporadic cases occurring in 2003 and in the two strains isolated in 1984. Probing using specific *int*2 and 16sRNA probes confirmed the chromosomal location of both types of class 2 integrons (see additional file 2).

All isolates belonging to PFGE types B and C proved to possess the 2161 bp class 2 integron. Moreover, a closest association was found between pulsotype A and the 1371 bp class 2 integron: indeed, all, but one, PFGE type A isolates were found to carry the smaller class 2 integron (Additional file 1 and Figure 1).

## Discussion

Our study was undertaken to perform a baseline assessment of the epidemiologic features characterizing the endemic circulation of *S. sonnei *in Iran.

Biotype g included all, but three, recent isolates of *S. sonnei*. Emergence of biotype g or rhamnose negative strains has been repeatedly signaled in several countries from different continents in association with the characteristic resistance pattern streptomycin-trimethoprim frequently associated to tetracycline [4,8,11]. Moreover, antimicrobial susceptibility testing of the Iranian isolates showed a highest prevalence of a consistent resistance profile, accordingly to the reports from a number of countries [3,6,28-31]. These antibacterial drugs are commonly used worldwide in the treatment of dysentery. No resistance was observed to ciprofloxacin, ceftriaxone, and chloramphenicol, probably because of their very infrequent use as alternative therapeutic regimens in our geographic area. Selective pressure by indiscriminate use of antibiotics and mechanisms of horizontal gene transfer have undoubtedly contributed to the emergence and diffusion of multi- resistance in *Shigella *[6].

Although other mechanisms are possible, resistance to streptomycin and trimethoprim seems to be attributable to expression of genes contained in class 2 integrons. These structures have proved to be highly prevalent within isolates of *Shigella *spp. from a number of countries [4,6,8,10,11,14]. Their gene array, generally including the open reading frames *dhfrI*, *sat *and *aad*, is rather stable, because of the presence of a defective integrase, but some different structures have been very infrequently found in sporadic isolates of *S. sonnei *[3,8,32]. Our paper firstly describes the simultaneous circulation at approximately similar frequencies (49.1% vs. 42.1%) in an apparently endemic context of two groups of strains carrying, respectively, a class 2 integron of 2161 bp and a shorter integron, not containing the *aad *gene cassette sequence. No specific association was apparent with the resistance pattern.

When considering results obtained by PFGE, a first impressive feature is the limited genetic heterogeneity of the isolates under study, along with their clustering in a few predominant PFGE types. These findings could suggest that what is apparent as an endemic circulation of *S. sonnei *in Tehran, Iran, is more likely to be interpreted as an oligo-clonal epidemic. The paucity of types for a pathogen which has only humans as a significant reservoir of infection, might derive from few sources of infection in a context of epidemiological transition that, along with the recent switching of predominant serogroup from *flexneri *to *sonnei*, could suggest increasingly effective sanitary conditions in Tehran, Iran. On the other hand, success of *S. sonnei *is warranted also in developed countries by low infectious minimal dose, high secondary attack rates and, in comparison with the other serogroups, a better capability to survive to stressing environmental conditions and exploit alternative and more complex infectious routes, such as food vehicles [33-35].

When considering the association of phenotypic and genetic properties of multidrug resistance, our findings reinforce the previous considerations and suggest the involvement of our geographic area within an epidemiological global picture of emergence and/or dissemination of a limited number of well defined multiresistant clones of *S. sonnei*. The question whether a multicentric emergence under analogous selective factors of similar particularly "fit" organisms or, alternatively, a dissemination driven by the international trade of human and food related resources were the most scientifically sound mechanism, arises with special evidence, when considering the geographic scale of the phenomenon.

Molecular epidemiology provides a crucial contribution to accurately interpret epidemiological evolution of infectious diseases in communities, when screening by phenotypic methods, such as biotyping or drug resistance pattern analysis, is seriously hindered by the homogeneity of circulating strains.

## Conclusion

The results suggest that biotype g, class 2 integron carrying *S. sonnei *are prevalent in our geographic area. *S. sonnei *isolated in the years 2002 and 2003 could be attributed to a few predominant clusters including, respectively, strains with pulsotypes B and C carrying a 2161 bp class 2 integron, and those having pulsotype A and a 1371 bp class 2 integron. A few epidemic clones are responsible for the apparently endemic occurrence of shigellosis in Tehran, Iran.

## Competing interests

The author(s) declare that they have no competing interests.

## Authors' contributions

RR and NS conceived the study, carried out biotyping, drug resistance analysis and drafted the manuscript. RR and AA performed PFGE analysis and detection of class 2 integrons. GMG carried out analysis of genetic similarity. AMD performed hybridization and sequencing experiments. CM participated in study design and coordination and helped to draft the manuscript. All authors read and approved the final manuscript.

## Pre-publication history

The pre-publication history for this paper can be accessed here:



## Supplementary Material

Additional file 1 Characteristics of *S. sonnei *isolates identified in Tehran, Iran, 2002–2003 Click here for file

Additional file 2 PFGE-*Ceu*I and hybridization of representative isolates of *S. sonnei*. DNA restriction was done with 10U *Ceu*I enzyme (New Englad Biolabs, Beverly, MA, US) at 37°C for 3 h and DNA macrorestriction fragments were resolved on 0.7% agarose gel (Pulsed Field Certified, Bio-Rad, Hercules, CA, US) suspended in 0.5X TBE buffer (50 mM Tris, 50 mM boric acid, 0.5 mM EDTA). Lambda ladder concatamers (New England Biolabs) were used as molecular marker. The gel was run on a CHEF-DRII system (Bio-Rad Laboratories) under the conditions described by Liu *et al*. (1993). The gel was stained for 10 min in ethidium bromide solution (0,4 μg/ml), destained in distilled water for 20 min, then visualised under UV light and photographed. Restricted fragments were transferred onto positively charged nylon membranes (Roche Diagnostics, Monza, Italy) by standard methods (Southern, 1975). Southern blot hybridization was carried out under high-stringency conditions, by using specific *int*2 and 16sRNA probes obtained amplifying the internal portion of the respective genes and labelling the probes by the "PCR DIG labelling" kit (Roche Diagnostics). Click here for file
