# Supplementary material for: Genetic relatedness among isolates of Shigella sonnei carrying class 2 integrons in Tehran, Iran, 2002–2003
Source: BMC Infect Dis. 2007 Jun 22;7:62. doi: 10.1186/1471-2334-7-62 (PMC1914347; doi:10.1186/1471-2334-7-62)
Supplement: Additional file 1 — Characteristics of S. sonnei isolates identified in Tehran, Iran, 2002–2003 [file 1471-2334-7-62-S1.doc]

**TABLE 1.** Characteristics of *S. sonnei* isolates identified in Tehran, Iran, 2002-2003

________________________________________________________________________________________________________

|  |  | Class 2 integron | Biotype | Antibiotic resistance* | | | | | | | | |  |
| --- | --- | --- | --- | --- | --- | --- | --- | --- | --- | --- | --- | --- | --- |
| PFGE pattern | |  |  |  |  |  |  |  |  |  |  |  | No. of |
| type | subtype |  |  | AMP | C | CP | CRO | NDA | K | STR | SXT | TET | strains |
| A | a | L | g | S | S | S | S | S | S | R | R | R | 17 |
|  | a | L | g | S | S | S | S | S | S | S | R | R | 1 |
|  | b | L | g | S | S | S | S | S | S | R | R | R | 5 |
|  | b | X | g | S | S | S | S | S | S | R | R | R | 1 |
|  | c | L | g | S | S | S | S | S | S | S | R | S | 1 |
| B | a | H | g | R | S | S | S | S | S | R | R | R | 4 |
|  | a | H | g | R | S | S | S | R | S | R | R | R | 1 |
|  | b | H | g | S | S | S | S | R | S | R | R | R | 2 |
| C | a | H | g | S | S | S | S | S | S | R | R | R | 12 |
|  | a | H | g | S | S | S | S | S | R | R | R | R | 2 |
|  | a | H | g | S | S | S | S | R | S | R | R | R | 1 |
|  | b | H | g | S | S | S | S | S | S | R | R | R | 5 |
|  | c | H | g | S | S | S | S | S | S | R | R | S | 1 |
| D |  | X | g | ND | ND | ND | ND | ND | ND | ND | ND | ND | 1 |
| E | a | X | g | ND | ND | ND | ND | ND | ND | ND | ND | ND | 1 |
|  | b | X | g | ND | ND | ND | ND | ND | ND | ND | ND | ND | 1 |
| F |  | ND | ND | S | S | S | S | S | S | S | S | S | 1^†^ |
| G | a | L | a | S | S | S | S | S | S | R | R | R | 1 |
|  | b | L | a | S | S | S | S | S | S | R | R | R | 1 |
| H |  | H | a | S | S | S | S | S | S | R | R | R | 1 |

* AMP, ampicillin, chloramphenicol; CP, ciprofloxacin; CRO, ceftriaxone; NDA, Nalidixic acid; K, kanamycin; STR, streptomycin ; SXT:trimethoprim- sulfamethoxazole; TET: tetracycline; R, resistant; S, susceptible; ND, not determined; X = negative for class 2 integron,^†^ ATCC 92
