# Supplementary figures and images for: Genetic relatedness among isolates of Shigella sonnei carrying class 2 integrons in Tehran, Iran, 2002–2003
Source: BMC Infect Dis. 2007 Jun 22;7:62. doi: 10.1186/1471-2334-7-62 (PMC1914347; doi:10.1186/1471-2334-7-62)

## Slide 1
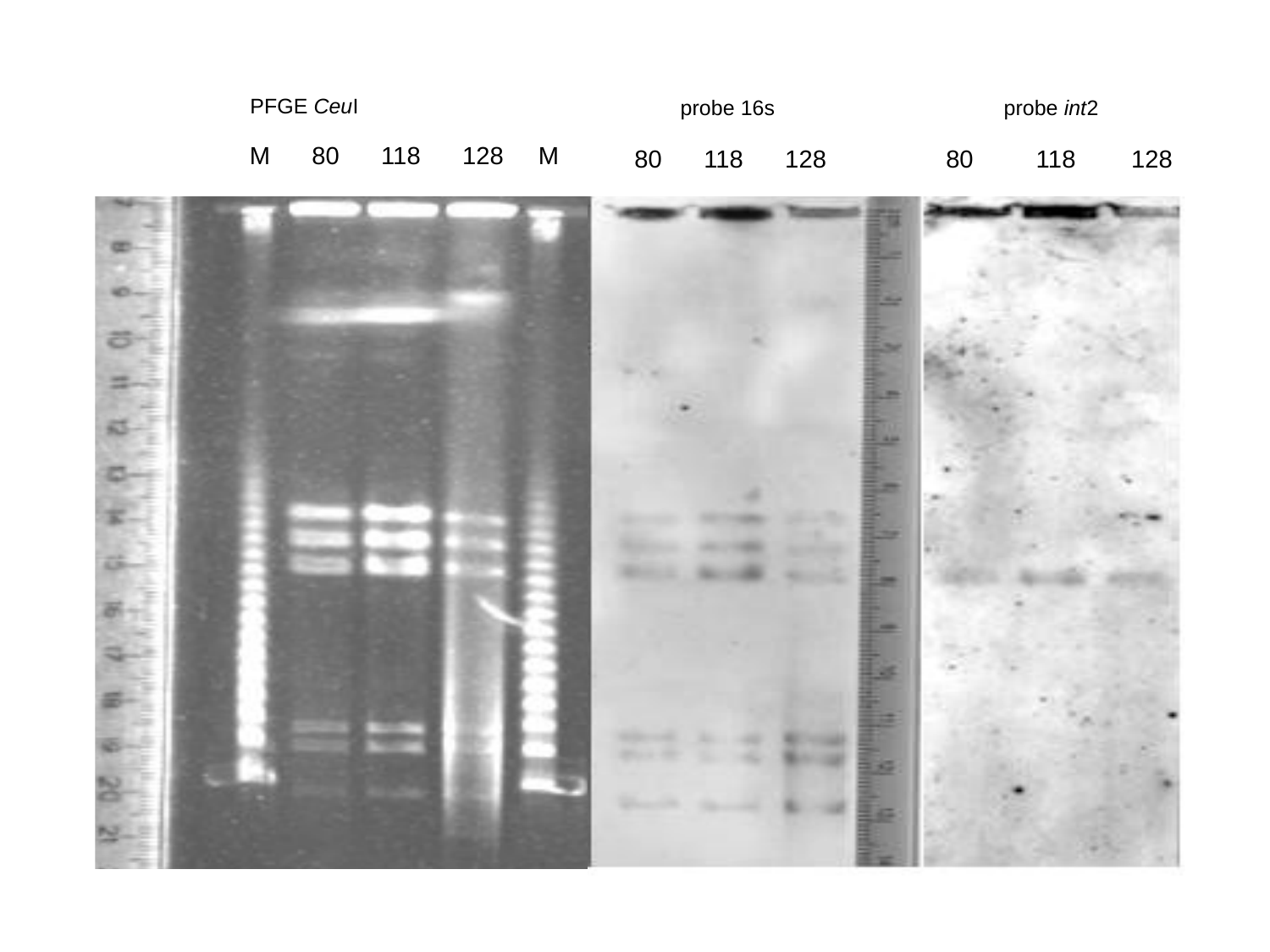

PFGE CeuI
 probe 16s
 probe int2
 M 80 118 128 M
 80 118 128
 80 118 128

Supplement: Additional file 2 — PFGE-CeuI and hybridization of representative isolates of S. sonnei. DNA restriction was done with 10U CeuI enzyme (New Englad Biolabs, Beverly, MA, US) at 37°C for 3 h and DNA macrorestriction fragments were resolved on 0.7% agarose gel (Pulsed Field Certified, Bio-Rad, Hercules, CA, US) suspended in 0.5X TBE buffer (50 mM Tris, 50 mM boric acid, 0.5 mM EDTA). Lambda ladder concatamers (New England Biolabs) were used as molecular marker. The gel was run on a CHEF-DRII system (Bio-Rad Laboratories) under the conditions described by Liu et al. (1993). The gel was stained for 10 min in ethidium bromide solution (0,4 μg/ml), destained in distilled water for 20 min, then visualised under UV light and photographed. Restricted fragments were transferred onto positively charged nylon membranes (Roche Diagnostics, Monza, Italy) by standard methods (Southern, 1975). Southern blot hybridization was carried out under high-stringency conditions, by using specific int2 and 16sRNA probes obtained amplifying the internal portion of the respective genes and labelling the probes by the "PCR DIG labelling" kit (Roche Diagnostics). [file 1471-2334-7-62-S2.ppt]
